# Overcoming adaptive resistance in non-small cell lung cancer: integrating targeted therapies, immunotherapy synergy, and nanocarrier delivery

**DOI:** 10.3389/fphar.2026.1960752

**Published:** 2026-10-16

**Authors:** Luke Ding, Anna Staskiewicz, Jesse Kao, Xinyu Wang

**Affiliations:** 1 Lambert High School, Suwanee, GA, United States; 2 Biochemistry, Cell and Developmental Biology Graduate Program, Emory University, Atlanta, GA, United States; 3 Doctor of Osteopathic Medicine Program, Philadelphia College of Osteopathic Medicine - Georgia Campus, Suwanee, GA, United States; 4 Department of Biomedical Sciences, Philadelphia College of Osteopathic Medicine – Georgia Campus, Suwanee, GA, United States

**Keywords:** combination therapy, nanocarrier delivery, immunotherapy, lung cancer, pharmacological synergy, targeted therapy, tumor resistance, tumor microenvironment

## Abstract

Lung cancer is the leading cause of cancer death, characterized by high mortality rates, tumor adaptation and heterogeneity, and immune evasion mechanisms. Despite recent advancements in chemotherapy, radiation, and surgery, survival rates for non-small cell lung cancer (NSCLC) and small cell lung cancer (SCLC) remain low, signifying the need for new strategies and innovative treatments. This review explores the combinatorial potential of immunotherapy with direct targeting of tumor growth to address the multifaceted challenges of NSCLC. Key approaches include small-molecule inhibitors, programmed death-ligand 1 (PD-L1) inhibition, angiogenesis inhibition, and nanotechnology-based drug delivery systems. Nanocarrier systems enable coordinated drug release and pharmacological synergy, thereby ensuring effective inhibition of pathways in the tumor microenvironment (TME). Additionally, this review addresses several resistance mechanisms in the context of specific inhibitors (e.g., EGFR and ALK inhibitors). Translational insights help bridge preclinical findings to clinical applications with an emphasis on angiogenesis inhibition and personalized approaches. Ethical considerations such as consent and preclinical safety testing are emphasized to ensure the safe development of these therapies. With the new generation of genetic engineering, immunotherapy, and targeted therapies for tumor growth, this work discusses the potential to transform lung cancer treatment.

## 1 Introduction

### 1.1 Significance of lung cancer

Lung cancer is one of the deadliest forms of cancer. This malignancy causes almost 125,000 deaths per year in the US, nearly 3 times as many as colorectal cancer, the second most common cause of cancer death (Bray et al., 2024; Siegel et al., 2025). Lung cancer lethality is partially attributed to delayed detection and diagnosis due to immune evasion and tumor heterogeneity (McPherson et al., 2020; Han et al., 2024). It is categorized into small cell lung cancer (SCLC) and non-small cell lung cancer (NSCLC), with NSCLC accounting for 80%–85% of all lung cancer cases (Papavassiliou et al., 2025; Alduais et al., 2023). NSCLC, subcategorized into squamous-cell carcinoma, adenocarcinoma, and large-cell carcinoma, originates in the peripheral lung tissue, while SCLC originates in the bronchi. SCLC is more aggressive than NSCLC and is characterized by rapid proliferation and early metastasis (Alduais et al., 2023). Furthermore, NSCLC cells can transform into SCLC, complicating clinical treatment options (Yang and Fan, 2024). Due to these distinctions, these cancers require separate therapeutic strategies and present unique clinical challenges. Despite recent advances in chemotherapy, radiation, and targeted therapies, survival outcomes for patients with lung cancer remain low, with a 5-year relative survival rate of 27% from 2014 to 2020 in the US (Siegel et al., 2025). This review primarily focuses on NSCLC-targeting treatments given their prevalence.

Historically, lung cancer, like other solid cancer counterparts, was mostly treated with radiation, chemotherapy, and surgical resection. The 1940s marked the introduction of nitrogen mustards into cancer chemotherapy, followed in the subsequent decade by advances in radiotherapy with the adoption of megavoltage linear accelerators (Jaklitsch et al., 1995). The development and application of new chemotherapeutic agents prolonged patient survival, such as the transition from platinum-based agents to taxanes and gemcitabine in the 1990s (Bunn and Kelly, 1998). Treatment paradigms have changed in recent decades, especially with the advent of immunotherapies such as immune checkpoint inhibitors (Robert, 2020). However, tumor resistance remains a prevalent concern, demonstrating a need for combination therapy and pharmacological synergy in treatment options.

### 1.2 Rationale to review treatment options

Recently, lung cancer treatments have shifted from traditional chemotherapeutic approaches to increased use of immunotherapy and targeted therapy, leading to advancements in clinical oncology (Wang et al., 2022a; Xiang et al., 2024). This is due to a growing understanding of lung tumor-immune system interactions, particularly the establishment of the tumor microenvironment (TME). TME is a tumor-promoting, immunosuppressive niche that recruits leukocytes to enable immune surveillance evasion (Racacho et al., 2025). Therefore, there has been a push to ensure that current strategies and preventive measures suppress tumor growth and metastasis while preventing tumor immune evasion and leukocyte recruitment (Racacho et al., 2025; Aliazis et al., 2025). While single-agent therapies can target key mutations in malignant cells, they often fall short due to tumor heterogeneity and adaptive resistance via secondary mutations, alternative signaling pathways, or immune cell recruitment.

Emerging cytokine therapies, nanotechnology-based drug delivery systems, and immune checkpoint inhibitors can offer improved efficacy and reduced non-specific toxicity (Sharma et al., 2023; Rawal and Patel, 2021; Xu-Monette et al., 2017; Chen et al., 2019; Essogmo et al., 2023). Preclinical studies lay the foundation for these innovations, revealing key mechanisms of tumor resistance and immune evasion that have now been translated into clinical applications (Wang et al., 2022a; Kang et al., 2024). There are several ways tumors can evade immune surveillance, such as by increasing the expression of programmed death-ligand 1 (PD-L1) to prevent T cell activation. Importantly, this can induce resistance to chemotherapies that rely on this pathway to proliferate T cells (Said and Ibrahim, 2023; Xu-Monette et al., 2017; He et al., 2015). With the integration of molecular insights from laboratory models and patient-centered therapies, lung cancer treatment is moving towards more personalized, adaptive approaches. This review aims to integrate these developments, emphasizing the importance of combined immune recovery and tumor-targeting as a synergistic treatment strategy. These approaches address the multipronged, resistant nature of lung cancer while optimizing patient-specific therapeutic windows.

## 2 Causation and pathogenesis of lung cancer

### 2.1 Etiological factors

Lung cancer incidence has decreased since 1984, yet it remains the primary cause of cancer-related deaths, resulting in 1.8 million deaths globally in 2020 (Zhou et al., 2024; Sung et al., 2021). While lung cancer prevalence is attributed to a multitude of factors, smoking is the prominent cause. Cigarette smoke contains more than 7,000 chemicals, including approximately 80 compounds classified as carcinogenic, such as benzene, arsenic, and cadmium (Li and Hecht, 2022; Rodgman and Perfetti, 2008). Smoking is also the leading cause of chronic obstructive pulmonary disease (COPD), a common comorbidity of lung cancer characterized by inflammation and damage to bronchi and alveoli (Zhou et al., 2023a). Secondhand smoke (SHS) exposure increases the risk of developing lung cancer by at least 20% in non-smokers (Possenti et al., 2024). The age of SHS exposure is a contributing factor, as people exposed before the age of 25 have increased lung cancer risk compared to those exposed later in life (Asomaning et al., 2008).

The second major contributing factor to lung cancer risk is air pollution, which includes particulate matter and gases such as ozone (Zhou et al., 2023a). Polycyclic aromatic hydrocarbons (PAHs) and n-nitroso compounds are major carcinogens in polluted air (Berg et al., 2023). Fine particulate matter (i.e., PM_2.5_), which contains high concentrations of PAHs, is of particular concern due to its ability to permeate deeply into lung tissue (Chen et al., 2025a). PM_2.5_ can increase inflammation and oxidative stress in the lungs and activate oncogenes (Chen et al., 2025a). Indoor air pollutants, including radon and asbestos, are also linked to lung cancer risk (Turner et al., 2020).

Inherited germline mutations contribute to lung cancer susceptibility. Pathogenic germline variants (PGVs) identified in lung cancer patients include *BRCA2* (breast cancer gene 2), *CHEK2* (checkpoint kinase 2), *ATM* (ataxia-telangiectasia mutated), *TP53* (tumor protein P53), and *EGFR* (epidermal growth factor receptor) (Sorscher et al., 2023). Li-Fraumeni syndrome, a cancer susceptibility syndrome associated with PGVs in *TP53*, increases the risk of lung adenocarcinoma and other cancers (Benusiglio et al., 2021). Specifically, patients with Li-Fraumeni syndrome and lung adenocarcinoma often present with acquired somatic *EGFR* mutations (Benusiglio et al., 2021). Somatic mutations in *EGFR*, *ALK* (anaplastic lymphoma kinase), and *KRAS* (Kirsten rat sarcoma virus) are frequently observed in NSCLC and are further discussed in the next section.

Inflammation can also play a key role in tumor formation. Chronic inflammation may arise from repetitive smoking or prolonged exposure to harmful chemicals, such as those in heavily industrialized areas, and forms a tumor-promoting environment with increased cytokines and accumulation of mutations (Gomes et al., 2014). Though macrophages and neutrophils are recruited to the lungs to remove harmful inhaled substances, they often kill key healthy cells and induce chronic inflammatory responses (Gomes et al., 2014; Aloe et al., 2021). Patients with the chronic inflammatory condition of COPD, which often presents with bronchitis and/or emphysema, have an increased risk of developing lung cancer (Wang et al., 2018). Neutrophil recruitment plays a primary role in COPD, as neutrophils are more prone to damage healthy cells than macrophages; such chronic inflammation contributes to lung cancer development (Gomes et al., 2014; Nolan et al., 2022; Aloe et al., 2021). Chronic inflammation can increase cell turnover in damaged epithelial tissue, increasing the likelihood of mutations through more frequent DNA replication. Neutrophils recruited to lung tissue can release reactive oxygen species (ROS) and proteases, which contribute to DNA damage (Wang et al., 2018).

### 2.2 Molecular basis of lung cancer development

Lung cancer is driven by complex interactions among genetic alterations, signaling pathways, and cellular division. *EGFR, KRAS*, *ALK*, and *ROS1* (ROS proto-oncogene 1, receptor tyrosine kinase) are oncogenic driver genes in lung cancer. Tumor suppressor genes, including *TP53* and *RB1* (retinoblastoma 1), play significant roles in tumor suppression and DNA stability. In a NSCLC patient screening, *KRAS* mutations, *EGFR* mutations, and *ALK* rearrangements were identified in ∼28%, ∼18%, and ∼4% of patients, respectively (Gainor et al., 2013). *EGFR* mutations, such as exon 19 deletions and L858R point mutations, can lead to constitutive activation of the tyrosine kinase EGFR. This drives oncogenic signaling through pathways including RAS/RAF/MAPK and PI3K/AKT/mTOR to promote aberrant cell proliferation (Figure 1). *EGFR* mutations are especially prevalent in non-smoking lung cancer patients and are targeted with tyrosine kinase inhibitors (TKIs), such as osimertinib (Gao et al., 2016; Borghaei et al., 2015).

**FIGURE 1:**
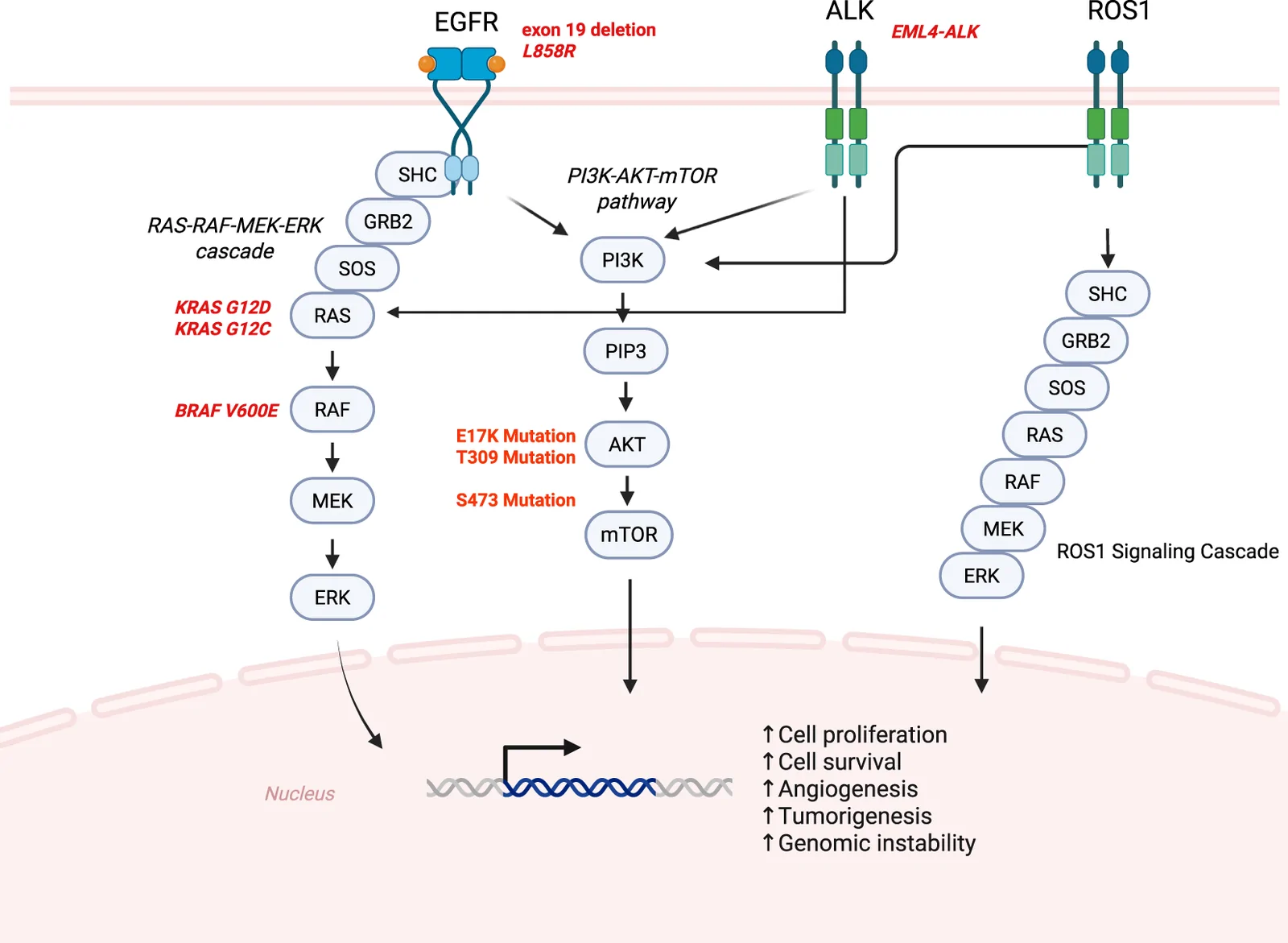
Common pathways contributing to cancer proliferation. Key mutations denoted in red. Created in BioRender. BioRender.com/lzwtvfe.

Furthermore, *KRAS* exhibits oncogenic properties similar to those of *ALK* (Alshammari et al., 2023; Kang et al., 2024). Activity of the GTPase, KRAS, is stimulated by activated tyrosine kinase receptors, including EGFR, ALK, and ROS1. Common *KRAS* mutations, including *KRASG12C* and *KRASG12D*, maintain KRAS in an active GTP state, thus promoting cell proliferation (He et al., 2025; Tang et al., 2024). *KRASG12D*-mutated NSCLC is associated with poorer responses to immune checkpoint inhibitors due, in part, to its inverse correlation with PD-L1 levels and reduced CD8^+^ tumor-infiltrating lymphocytes in the TME (Liu et al., 2022). *ALK* rearrangements, including the *EML4-ALK *fusion gene, are found in NSCLC patients and contribute to aberrant MAPK and PI3K signaling. *ALK*-positive tumors initially respond to ALK inhibitors such as alectinib and lorlatinib; however, resistance mechanisms in the tumor can cause secondary mutations to develop. Zhuang *et al.* screened samples from NSCLC patients for *EGFR, KRAS, ALK, ROS1,* and *BRAF* (B-Raf proto-oncogene, serine/threonine kinase) and found that 1.7% of samples had more than one gene mutation, with *EGFR* being the most commonly co-mutated gene (Zhuang et al., 2019).

Mutations in key tumor suppressor genes, such as *TP53* and *RB1*, accelerate disease progression by removing critical controls on cell cycle regulation. *TP53* mutations are also among the most common genomic alterations in lung cancer. *TP53* and *RB1* are often co-mutated, with concomitant mutation frequencies reported at ∼36% (Saghaeiannejad Esfahani et al., 2021). *EGFR/TP53/RB1* triple mutant lung cancers have a particularly poor prognosis compared to *EGFR/TP53* or *EGFR*-only mutants and are prone to histological transformation into SCLC (Offin et al., 2019).

Oncogenic and suppressor gene alterations affect treatment potential in variable ways. SCLC cells exhibit some form of resistance toward drugs that target the MAPK pathway. Several studies show that inhibition or alteration of PI3K-mTOR and BCL-2 pathways may contribute to SCLC development (Kang et al., 2024; Li et al., 2021). Furthermore, activation of pathways such as Notch and KRAS can dampen cancer cell response to chemotherapy by upregulating survival and proliferation signaling (Kang et al., 2024). Targeting these mechanisms with personalized therapies that address patient-specific tumor properties could modernize lung cancer treatment and remains an active area of research.

### 2.3 TME in lung cancer

The TME plays a central role in tumor progression and therapeutic resistance, comprising both cellular and non-cellular components that dynamically interact with cancer cells to either support or suppress tumor growth. Among these cellular components are key modulators of antitumor immunity: T cells, B cells, natural killer (NK) cells, macrophages, and myeloid-derived suppressor cells (MDSCs). However, tumors can recruit these cells (e.g., tumor-associated neutrophils and macrophages) to support their own survival. These cells are also influenced by cancer-associated fibroblasts and endothelial cells, which suppress immune cells in the TME (Lim et al., 2023; Alshammari et al., 2023; Wu et al., 2022) (Figure 2). Tumor-associated macrophages (TAMs) have tumor immunity properties, and, when paired with the CCL2 axis, can promote M2-type polarization where macrophages acquire the M2 anti-inflammatory and tissue repair phenotype. M2 is essential in suppressing excessive immune responses. With the addition of MDSCs, this could contribute to immunosuppression with the secretion of anti-inflammatory cytokines and suppression of T cell activity (Fung et al., 2021; Xiang et al., 2024; Kadomoto et al., 2021). While tumor-associated neutrophils can be reprogrammed to exert N1 antitumor effects, the potential for a protumor switch to N2 raises concerns about this treatment approach (Figure 2).

**FIGURE 2:**
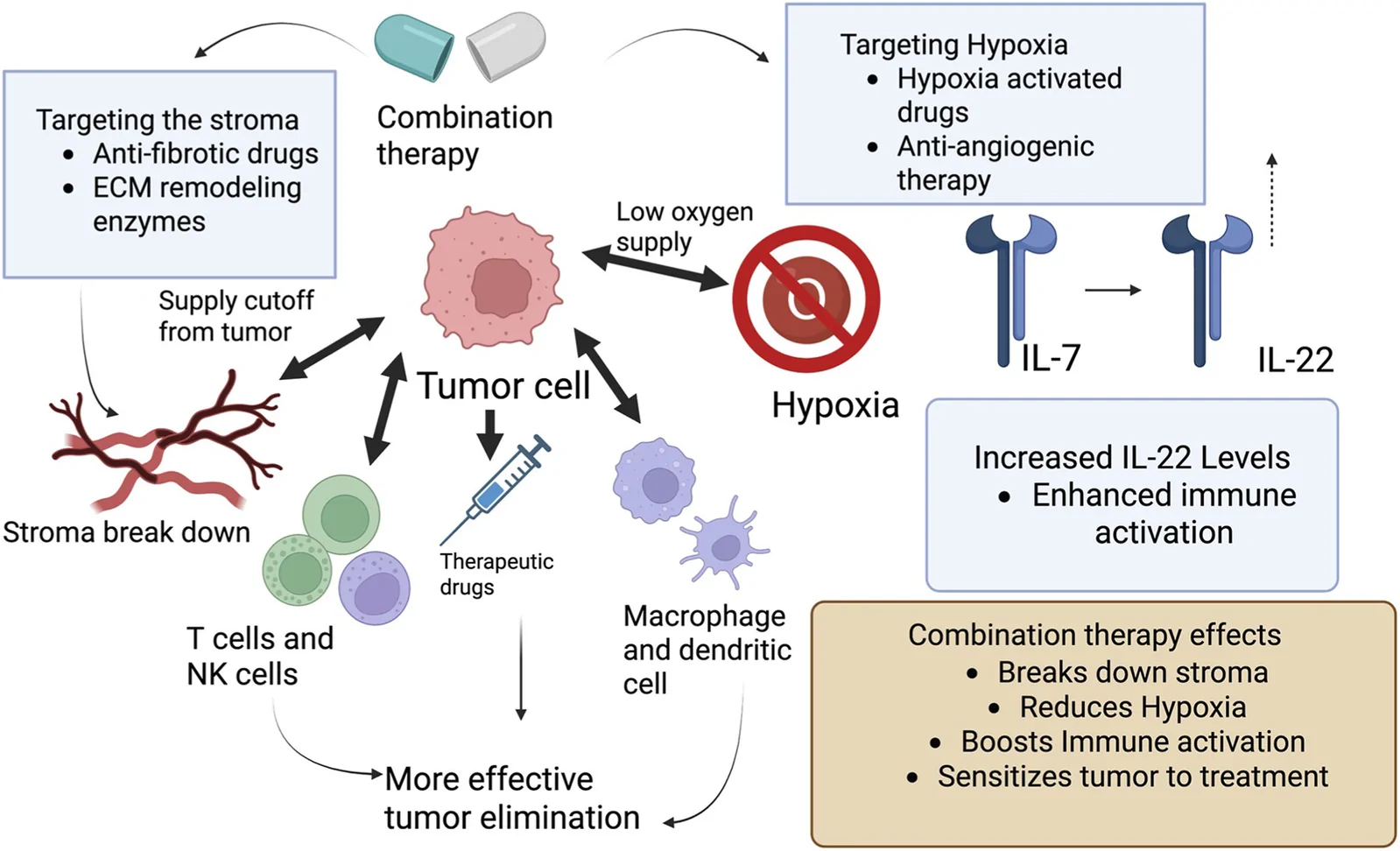
Combination therapies reprogram the tumor microenvironment by targeting stromal barriers and hypoxia, and by enhancing cytokine-driven immune activation (IL-7/IL-22). These effects increase T-cell and NK-cell infiltration, improve drug penetration, and sensitize tumor cells to immunotherapy, chemotherapy, and targeted treatments (Lim et al., 2023; Wu et al., 2022; Zhang et al., 2021). Created in BioRender. BioRender.com/m57v1qf.

Non-cellular components of the lung microenvironment, such as the extracellular matrix (ECM), cytokines, and chemokines, also play major roles in lung cancer progression, therapy resistance, and immune evasion. The ECM can be remodeled to better protect cancer cells from drug penetration and from cytotoxic lymphocytes, and IL-7 can mediate STAT5 activation to regulate T cell cytotoxicity and drug resistance (Wang et al., 2022a; Cheng et al., 2016) (Figure 2). Additionally, the cytokine interleukin-22 (IL-22) is expressed in macrophages and T cells in the lungs and has been shown to protect cancerous cell lines (Sharma et al., 2023; Yi et al., 2023). This effect supports tumor cell survival by inhibiting apoptosis and promoting angiogenesis (Xu et al., 2024). TGF-β is another mediator of the lung TME, as it is strongly associated with metastasis, tumor invasion, and immunosuppression (Zhou et al., 2018; Hourani et al., 2025). Furthermore, TGF-β regulates angiogenesis and ECM formation (Chung et al., 2021). TGF-β inhibitors may be administered in combination with other treatments due to TGF-β′s angiogenic, immunosuppressive, and tumor-supportive properties (Liu et al., 2026). Targeting these TME components in combination with directed therapies can function synergistically by enhancing immune activation and reducing pro-tumor signaling.

Preclinical studies have applied platforms such as *in vitro* 3D co-culture systems and *in vivo* models (e.g., patient-derived xenograft (PDX) mice) to dissect TME biology. These models have been used to analyze immune cell infiltration, PD-1 inhibition, cytokine crosstalk, and therapeutic responses (Xue et al., 2023; Veith et al., 2024; Oswald et al., 2022). For instance, reduced tumor volume and enhanced infiltration of PD-1^+^ T cells into the TME were observed following anti-PD-1 therapy in lung cancer models (Markowitz et al., 2018). Clinically, TME biomarker identification can guide immunotherapeutic development; examples include PD-L1 expression levels, cytokine signatures, and tumor-infiltrating lymphocytes (TILs) (Lim et al., 2023; Wong et al., 2022; Xiang et al., 2024).

## 3 Current treatment strategies for immune recovery and targeting tumor growth

### 3.1 Targeted therapies

Targeted therapies have transformed the treatment landscape for NSCLC by targeting specific oncogenic mutations. Several EGFR inhibitors have been used to treat patients with *EGFR*-positive NSCLC (e.g., osimertinib, erlotinib, gefitinib, afatinib, dacomitinib, and furmonertinib) (Zan et al., 2025). Fortunately, osimertinib treatment has increased progression-free survival (PFS) in cancer patients compared to earlier-generation inhibitors and is particularly effective against the T790M resistance mutation by inhibiting the tyrosine kinase activity of EGFR (Gao et al., 2016; Borghaei et al., 2015; Ramalingam et al., 2020; Gen et al., 2022). However, resistance mechanisms remain a challenge, as acquired mutations in genes such as *MET* (hepatocyte growth factor receptor) and *HER2* (human epidermal growth factor receptor 2) are increasingly targeted by next-generation inhibitors (Kang et al., 2024; Wang et al., 2022b). These mutations underscore the need for combination-based co-targeting strategies, as these pathways work alongside EGFR. PDX mouse models can be used to analyze the effectiveness of EGFR inhibitors and the development of resistance (Kita et al., 2019).

*ALK* and *ROS1* gene rearrangements are another subcategory of oncogenic drivers in NSCLC. Therapies such as alectinib, brigatinib, lorlatinib, and crizotinib are ALK/ROS1 inhibitors that could target these fusions, especially the *EML4-ALK* variant. These drugs have shown efficacy in *ALK*-driven models and have improved outcomes in patients with *ALK* rearrangements, including those with brain metastasis (Xiang et al., 2024; Gil et al., 2023; Ceddia and Codacci-Pisanelli, 2021). However, as with EGFR-targeted therapies, resistance often arises from secondary mutations or the activation of alternative signaling pathways, which underscores the need for a multi-targeted approach to cell-signaling blockade. ALK/ROS1 inhibitors such as alectinib act as ATP-competitive inhibitors, whereas brigatinib inhibits ALK by preventing phosphorylation (Xiang et al., 2024; Moon et al., 2020). *KRAS* mutations, on the other hand, were historically considered “undruggable” but have recently been effectively targeted by *KRAS* G12C inhibitors such as sotorasib and adagrasib (Figure 3). Clinically, these targeted therapies have FDA approval for *KRAS* G12C-mutant NSCLC and offer a more targeted approach than traditional chemotherapy (Nakajima et al., 2022; Tian et al., 2022). However, resistance via secondary mutations or pathway reactivation remains an issue in *KRAS*-mutant NSCLC and warrants further investigation into combination strategies.

**FIGURE 3:**
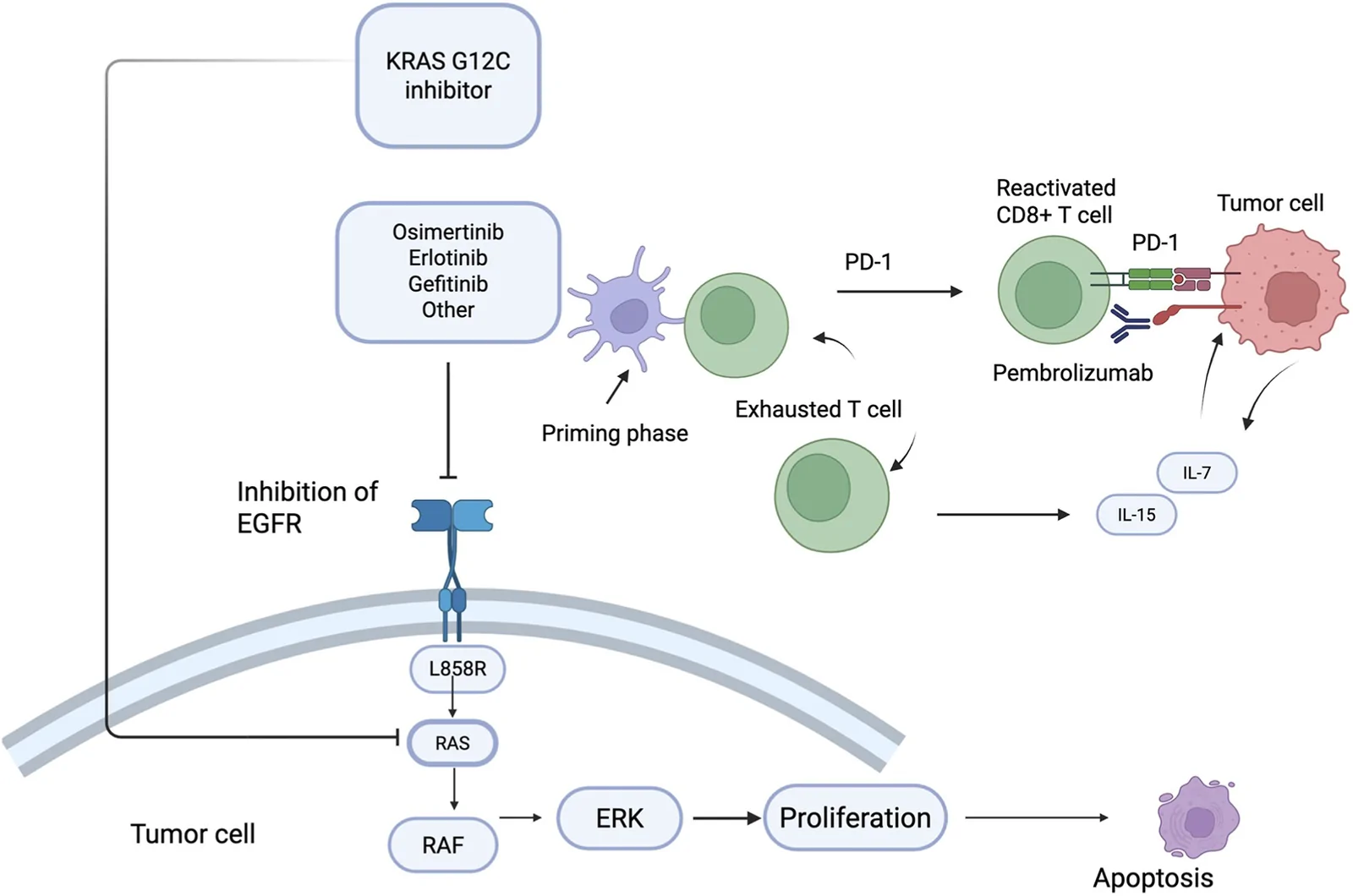
Combinatorial targeted therapy and immunotherapy as NSCLC treatment. Targeted drugs, such as those inhibiting EGFR or downstream targets like KRAS, induce tumor-specific cell death (Kang et al., 2024; Desai and Lovly, 2023), while PD-1 inhibition (via pembrolizumab) counteracts immune evasion and enhances CD8^+^ T cell function. Furthermore, IL-15 and IL-7 can help with activating additional T cells (Sharma et al., 2023; Khaddour et al., 2021). Created in BioRender. BioRender.com/ep8rpm9.

Other emerging targeted agents include inhibitors against *RET*, *MET*, *HER2*, and *BRAF* alterations. MET inhibitors, such as capmatinib and tepotinib, are approved treatments for patients with *MET* exon 14-skipping mutations (Paik et al., 2020; Mathieu et al., 2022). Furthermore, *HER2*-altered NSCLC, including *HER2* mutations and amplifications, has shown sensitivity to trastuzumab-based therapies and small-molecule HER2 inhibitors (Riudavets et al., 2021). Finally, the combinatorial therapy of BRAF and MEK inhibitors in *BRAF*-mutant-positive NSCLC results in improved response rates, particularly compared with conventional chemotherapy (Han et al., 2021).

### 3.2 Immunotherapies and immune checkpoint inhibitors

Immune checkpoint inhibitors have enabled new therapeutic strategies in lung cancer by restoring anti-tumor immune responses. Examples of effectiveness are seen in preclinical studies using syngeneic mouse models, which demonstrate their ability to reinvigorate exhausted T cells and improve tumor regression (Li et al., 2017; Markowitz et al., 2018). For instance, in a Lewis lung carcinoma mouse model, combining anti-PD-1 therapy with a dipeptidyl peptidase inhibitor increased T cell infiltration into tumors and reduced tumor growth compared with anti-PD-1 treatment alone (Lei et al., 2024). Clinically, PD-1 antibody treatment prolongs survival, especially in patients with high PD-L1 expression and tumor mutational burden (TMB) (Reck et al., 2021). PD-1 inhibitors (e.g., pembrolizumab, cemiplimab, and nivolumab) and PD-L1 inhibitors (e.g., durvalumab and atezolizumab) are standards of care for advanced NSCLC (Li et al., 2023b). Pembrolizumab is approved as a first-line monotherapy for patients with PD-L1 ≥ 50% or in combination with chemotherapy for the general population (Reck et al., 2021). *EGFR*-mutant tumors are associated with high PD-L1 expression, which correlates with poor prognosis and provides a rationale for a combination strategy targeting both pathways (Altorki et al., 2019; Akbay et al., 2013; Yang et al., 2019).

CTLA-4 inhibitors, such as ipilimumab, are combined with PD-1 inhibitors to enhance T cell activation at both the priming and effector phases, thereby providing additional B cell support. This dual blockade has shown effectiveness in preclinical models and benefits in clinical settings of NSCLC patients (Fridlender et al., 2011; Wang et al., 2025; Hosoya et al., 2026). However, immune-related adverse events such as colitis require caution (Ettl et al., 2022). Beyond PD-1 and CTLA-4, immune modulators such as TIGIT, LAG-3, and TIM-3 have been emerging as co-inhibitory receptors that could contribute to T-cell exhaustion, and their inhibition could improve outcomes when paired with existing immune checkpoint inhibitors (Alshammari et al., 2023; Xiang et al., 2024; Shen et al., 2018). Preclinical studies are underway to determine the optimal therapeutic window and the best combinations with checkpoint inhibitors. A recent study observed that a combination of a PD-1 antibody, chemotherapy, and a nanocarrier-delivered PDLIM2 (tumor suppressor) plasmid led to lung tumor remission in 60% of mice and increased CD4^+^/CD8^+^ T cell activation and infiltration, without increased toxicity from the nanoPDLIM2 (Sun et al., 2024). These strategies, when coordinated with immune response activation, oncogenic signaling suppression, and optimized drug delivery, can produce enhanced antitumor effects without increased toxicity.

The integration of immunotherapy into lung cancer treatment has enabled biomarker development and exploration of combination strategies, such as immunotherapy plus targeted therapies or chemotherapy, to overcome tumor resistance within the TME. Furthermore, using cytokine modulators like IL-7 and IL-15 could enhance T and NK cell function as well as enhance antigen responses in CD8^+^ T cells, which have shown promise in early trials (Wang et al., 2022a; Waldmann et al., 2020; Zhang et al., 2025; Cai et al., 2023).

## 4 Limitations of traditional treatment and recent updates on new therapeutic strategies

### 4.1 Current challenges with radiotherapy and chemotherapy

The current landscape of lung cancer has shifted rapidly from chemotherapy to more modern combination approaches. These advances aim to overcome the limitations of traditional treatments, such as first-generation inhibitors and radiotherapy, which are constrained by their targeting of healthy cells and by drug resistance. The new therapeutic strategies often improve tumor specificity, reduce toxicity, and enhance immune responses compared with their predecessors used for decades.

Radiotherapy and chemotherapy historically have been two of the main treatment options for lung cancer, though they present with considerable limitations. Chemotherapy induces systemic cytotoxicity by targeting proliferating normal tissues, leading to dose-limiting toxicity and a reduced therapeutic index. Radiotherapy, although localized, presents its own challenges. When radiation is used in high amounts, it could damage tumor DNA and release nuclear proteins like HMGB1, which can lead to further metastasis and inflammation (Wang and Zhang, 2020; Andersson and Tracey, 2011). This protein has been shown to contribute to tumor progression and pro-inflammatory cytokine release, leading to dendritic cell dysfunction and ultimately reducing T and B cell effectiveness (Zhang et al., 2019; Wu et al., 2015). Additionally, radiation therapy could cause cognitive decline and pulmonary fibrosis, which can worsen respiratory issues for patients (Knapp et al., 2022).

Importantly, cytotoxic chemotherapy and radiotherapy pose complex, bidirectional impacts on the TME (Chen et al., 2026). These approaches can stimulate anti-tumor immunity by inducing immunogenic cell death (ICD) and facilitating the release of tumor-associated antigens (TAAs) (Fabian et al., 2021). However, sublethal damage from radiation and chemotherapy frequently turns the TME into a highly immunosuppressive niche (Wei et al., 2026). Exposure to these approaches promotes the recruitment and polarization of pro-tumorigenic immune subsets, such as regulatory T cells (Tregs), myeloid-derived suppressor cells (MDSCs), and M2-like tumor-associated macrophages (TAMS) (Zhang et al., 2026; Becherini et al., 2023; Wang et al., 2024). Furthermore, chemotherapy that upregulates immune checkpoint molecules (such as PD-L1) and induces stroma remodeling can create physical and biochemical barriers to support immune evasion (Liu et al., 2025; Chen et al., 2025b).

Another limitation of both therapies is the high rate of tumor resistance. Tumors exposed to chemotherapy and radiation can undergo epithelial-to-mesenchymal transition (EMT), which allows cancer cells to evade immune detection and become more invasive in the lung TME (Duan et al., 2022). In addition, chemoresistant tumors can release chemoattractant cytokines such as CXCL12, also known as Stromal Cell-Derived Factor 1 (SDF-1), and activate immune-evasive pathways that diminish response to immunotherapies (Guyon, 2014; Zhou et al., 2014; Wang et al., 2023). For instance, the CXCL12/CXCR4 axis prevents CD8^+^ T cell infiltration into tumors (Singh et al., 2025). Tumor resistance to chemo- and radiotherapy is significantly driven by dysregulated miRNA expression, making specific microRNAs promising biomarkers for predicting treatment response and therapeutic targets for reversing resistance (Hummel et al., 2010). MicroRNAs play crucial roles in lung cancer progression, serving as dual-function regulators that act as either tumor suppressors or oncogenes. Consequently, specific miRNA expression profiles show strong potential as non-invasive diagnostic and prognostic biomarkers and as novel therapeutic targets, though clinical translation requires overcoming key delivery and stability challenges (Bartoszewska et al., 2025).

### 4.2 Emerging targeted therapies

Next-generation EGFR and ALK inhibitors have been developed to address acquired resistance seen in earlier drugs. Osimertinib, a third-generation EGFR-TKI, is currently recommended as a first-line therapy for *EGFR*-mutant lung cancer per the National Comprehensive Cancer Network (NCCN) guidelines and is particularly effective against the T790M mutation. It demonstrated enhanced CNS penetration, providing better control of brain metastases in *EGFR*-mutant NSCLC (Gao et al., 2016; Borghaei et al., 2015; Ramalingam et al., 2020). Similar to osimertinib, next-generation ALK inhibitors such as lorlatinib and alectinib demonstrate efficacy in overcoming secondary *ALK* mutations and achieving CNS activity (Shalata et al., 2025; Xiang et al., 2024). These therapies represent powerful approaches to targeting critical mutations, prolonging progression-free survival, and maintaining quality of life in patients with NSCLC.

Although EGFR and ALK remain among the most actionable drivers in NSCLC, expanding genomic insights have revealed additional molecular targets that are now being explored for their clinical relevance. Therapies targeting MET, such as capmatinib and tepotinib, have shown very promising results in patients with *MET* exon 14 skipping mutations in phase II clinical studies (Feldt and Bestvina, 2023). Other novel targets include TROP-2, HER3, and DLL3. TROP-2-directed antibody drug conjugate sacituzumab exhibits promising antitumor activity in advanced NSCLC, and HER3-targeted patritumab has advanced to early-phase trials (Riudavets and Planchard, 2025). In SCLC, DLL3, a protein overexpressed in tumor cells but not in healthy cells, is targeted by bispecific T-cell engagers (BiTEs) such as Tarlatamab (Ahn et al., 2023). Additionally, DLL3 targeting shows promise in addressing neuroendocrine transformation, a key mechanism of acquired resistance in which NSCLC transforms into a SCLC phenotype (Aredo et al., 2025). These targeted therapies are increasingly used as components of a synergistic multi-pathway blockade designed to prevent reactivation of compensatory signaling.

### 4.3 Advanced immunotherapeutic approaches

Recent advances in immunotherapy have expanded treatment options for lung cancer by adding strategies such as bispecific antibodies, antibody-drug conjugates (ADCs), CD8^+^ T-cell and NK-cell activation, and cytokine-based immune modulation. Bispecific antibodies, such as amivantamab, can target *EGFR* and *MET* mutations, providing a dual blockade of key resistance pathways and facilitating tumor eradication (Figure 4). These mechanisms underscore the importance of using dual-target immunotherapy to address the various mechanisms by which tumor resistance can arise in NSCLC (Zhou et al., 2023b). This demonstrates pharmacological synergy by simultaneously blocking multiple pathways, providing an amplified antitumor effect beyond those of individual treatments. Such combination strategies show clinical efficacy in trials, such as the phase III MARIPOSA trial, which evaluated the effects of bispecific antibody amivantamab with TKI lazertinib in *EGFR*-mutated NSCLC (Cho Byoung et al., 2024). Additionally, for SCLC, treatment with tarlatamab (a DLL3-targeting BiTE) showed promising survival outcomes in several clinical trials (Ahn et al., 2023; Mountzios et al., 2025).

**FIGURE 4:**
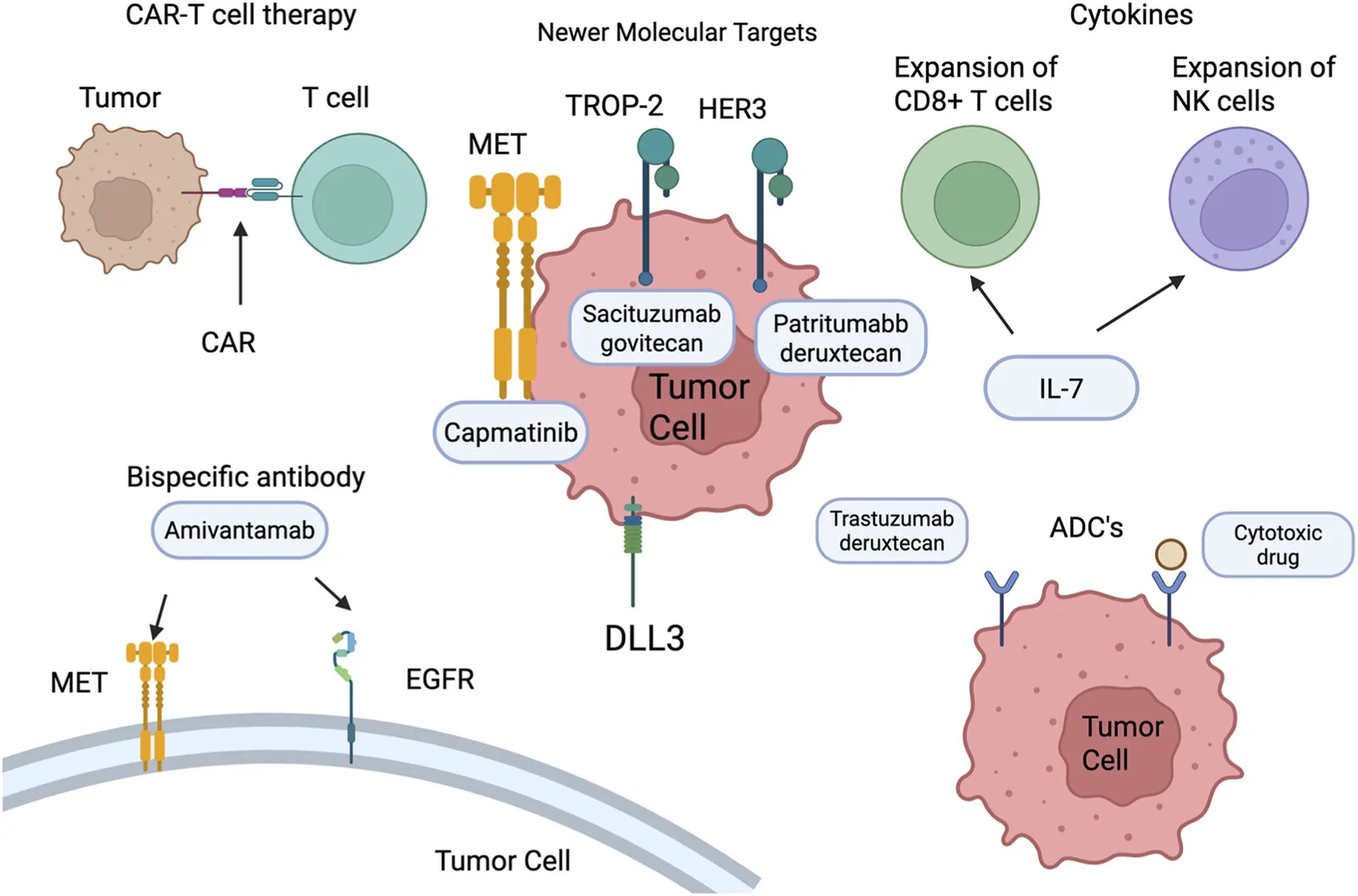
Advanced immunotherapeutic approaches. Modern approaches include CAR-T cell therapy, antibody-drug conjugates, bispecific antibodies like amivantamab, and cytokine modulation with IL-7, along with their processes (Li et al., 2024; Wang et al., 2022a; Luo et al., 2026). This figure also highlights new molecular targets and treatments in lung cancer (Gao et al., 2016; Gen et al., 2022; Stevens et al., 2020; Feldt and Bestvina, 2023; Ahn et al., 2023). Created in BioRender. BioRender.com/hkk5ujn.

ADCs have also reshaped lung cancer therapy by combining cytotoxic drugs with monoclonal antibodies that target tumor-specific antigens (Figure 4). Trastuzumab deruxtecan, approved for *HER2*-positive NSCLC, demonstrates how ADCs can improve specificity and efficacy while reducing off-target effects for this class of drugs (Li et al., 2024). Furthermore, patritumab deruxtecan and sacituzumab govitecan target HER3 and TROP-2, respectively, and are currently in clinical trials in NSCLC (Stumpo et al., 2025; Yu et al., 2023).

Although CAR-T cell therapy has been predominantly effective in hematologic malignancies, it is currently being explored for lung cancer (Figure 4). Even though there are challenges, such as limited infiltration into tumors and a suppressive TME. Genetic reprogramming of immune cells, such as engineering CAR-T cells to express chemokine receptors or resist TGF-β suppression, has enhanced TME infiltration and persistence in preclinical models (Ma et al., 2023). Similarly, tumor-infiltrating lymphocyte therapy has shown efficacy in early trials for refractory tumors (Creelan et al., 2021) and could be combined with cytokines to further enhance the immune response.

Cytokine-based immune modulation has remained an active area of research to counter TME-mediated immunosuppression. IL-7 and IL-15 analogs could boost CD8^+^ T cell and NK cell expansion and function (Figure 4). This is critical as these cells play key roles in enhancing immune memory and antitumor activity. As CD8^+^ T cells can indirectly influence B cell activation, this approach can further support anti-tumor responses. These effects have been demonstrated in preclinical settings and are now being considered in combination therapy trials evaluating cytokine superagonists alongside immune checkpoint inhibitors. (Wang et al., 2022a; Waldmann et al., 2020; Kielsen et al., 2021; Cai et al., 2023).

### 4.4 Combination therapies

More recently, the combination of multiple therapies has become an increasingly important strategy in lung cancer treatment. It is driven by advances in precision medicine and a growing understanding of tumor-adaptive resistance mechanisms and feedback-driven signaling networks. This approach is enhanced by pharmacological synergy in which combinations produce effects greater than the sum of their individual components, particularly by disrupting oncogenic signaling and immune evasion pathways. An example of this can be seen in targeted therapy and immunotherapy combinations, where direct inhibition of oncogenic mutations is paired with immune checkpoint blockade, enabling both tumor-intrinsic suppression and immune system reactivation in a synergistic manner rather than multiple scattered additive effects.

In current clinical practice, NCCN guidelines recommend platinum-based chemotherapy combined with immune checkpoint inhibitors as a first-line therapy for patients with advanced NSCLC who lack driver mutations. Such combinations show enhanced survival outcomes, as in the KEYNOTE-189 trial, which demonstrated prolonged overall survival from pembrolizumab plus platinum-based chemotherapy compared to chemotherapy alone (Gandhi et al., 2018). Importantly, the improved outcomes observed with these regimens are increasingly attributed to synergy between cytotoxic chemotherapy and immune activation, in which chemotherapy-induced tumor antigen release enhances the efficacy of immune checkpoint inhibitors by increasing tumor immunogenicity (Principe et al., 2022). Together, these strategies enable tailored clinical treatment for each patient based on the tumor’s immune context and oncogenic targets, potentially improving response rates while reducing toxicity in the TME.

Beyond immune-chemotherapy procedures, combining angiogenesis inhibition with targeted therapy or checkpoint blockade represents another synergistic approach. Agents targeting vascular endothelial growth factor (VEGF), such as bevacizumab, not only normalize tumor vasculature to enhance drug delivery but also relieve VEGF-mediated immunosuppression in the TME (de Aguiar and de Moraes, 2019). Phase III trials, such as RELAY (erlotinib plus ramucirumab) and IMpower150 (atezolizumab plus bevacizumab and chemotherapy), have validated the clinical relevance of dual angiogenesis and oncogenic/immune pathway blockade in improving progression-free survival (Kawczak and Bączek, 2026; Nakagawa et al., 2025; Socinski et al., 2021). Additionally, studies exploring VEGFR TKIs (e.g., fruquintinib and anlotinib) combined with PD-1 inhibitors show that restoring functional tumor perfusion enhances effector T-cell infiltration, shifting TMEs into immune-responsive environments (Fan et al., 2022; Li et al., 2022).

Combination therapy has been a central strategy in precision medicine for lung cancer with the aim of overcoming resistance mechanisms while enhancing therapeutic efficacy through biomarker-driven approaches. For instance, combining osimertinib, a third-generation EGFR TKI, with PD-1/PD-L1 inhibitors could yield antitumor effects, particularly in tumors with *EGFR* mutations and prolonged PD-L1 expression (Gao et al., 2016; Borghaei et al., 2015). Additionally, therapies that integrate KRAS or MET inhibitors with checkpoint blockade agents are currently in preclinical trials. Furthermore, fourth-generation ALK-TKI inhibitors such as TPX-0131 have been used to combat ALK-TKI resistance mechanisms in cancer stem cells. These combination therapies are very promising for treating tumors that have escaped immune checkpoint mutations, which reduce the efficacy of monotherapy (Kang et al., 2024; Desai and Lovly, 2023).

Biomarkers, such as TMB, TILs, and CD8^+^ T cell activity, are beginning to be used as indicators of tumor-immune interaction dynamics, allowing for the prediction of combination therapy responsiveness and potential pharmacological synergy (Lim et al., 2023; Kim et al., 2021; Wong et al., 2022; Xiang et al., 2024). As our knowledge of the TME and its interactions deepens, combining genetic and immune profiling will become a key part of personalized therapy by helping to overcome resistance and reduce both risk and relapse in patients.

## 5 Challenges in lung cancer treatment

### 5.1 Ethical considerations

One of the biggest ethical issues in lung cancer research is ensuring informed consent for experiments and the use of genomic data. Genomic data are inherently identifiable and highly personal because an individual’s genetic sequence is unique and can serve as a persistent biological identifier (Karlsson Linnér and Jain, 2025). Patients must be clearly informed about the risks, potential benefits, and long-term hazards associated with interventions. Other problems can include the confidentiality of patient data. Ensuring that personally identifiable data remains contained is key to preventing the unauthorized release of sensitive information (Allmark and Tod, 2016). In the context of precision oncology and pharmacologically synergistic therapies, genomic data are becoming increasingly sensitive because they are used directly to predict patient-specific drug response.

Additionally, equitable patient selection for therapies such as IL-7 or NK cell and CD8^+^ T cell manipulation has to account for multiple factors, such as tumor heterogeneity, environmental exposure (via smoking or air pollution), socioeconomic factors, and genetic disorders that can influence responses to treatment (Wang et al., 2022a; Asomaning et al., 2008; Shehata et al., 2025). Equitable patient selection must also address racial disparities in clinical trials. Historically, pharmacological and oncology trials have had disproportionately few participants of non-European ancestry, resulting in datasets that fail to reflect the global genetic pool. This lack of representation introduces systematic bias in pharmacological prediction models and limits the reliability of identified drug synergies across populations. This limitation is especially critical in synergy-driven pharmacology, where optimal drug combinations depend on nonlinear, patient-specific molecular interactions rather than single-agent effects. In the context of precision medicine, this issue is even more pivotal as strategies are increasingly guided by genomic data (Jamalinia and Weiskirchen, 2025). If genomic data lack diversity, therapy can be optimized only for a specific population, reducing its overall effectiveness. To mitigate this, research should prioritize developing diverse genetic databases that include patients from varied racial, ethnic, and socioeconomic backgrounds. Greater representation improves biomarker discovery and enhances the prediction of drug response. Furthermore, computational pharmacology and machine learning models are increasingly used to predict patient-specific pharmacological synergies and define individualized therapeutic windows, reinforcing the ethical necessity of diverse datasets for unbiased therapeutic optimization. Although ethical frameworks help ensure that emerging therapies are applied responsibly, the clinical effectiveness of these treatments remains limited by tumor drug resistance.

These ethical challenges require not only technical safety but also clear policies that respect lab safety and patient autonomy. The continual integration of bioethics into research and clinical treatment development ensures that therapies will not only become more personalized and effective but also be developed in ways that ensure patients feel safe and protected.

### 5.2 Overcoming drug resistance

Another challenge in lung cancer therapy, especially in NSCLC, is tumor resistance. Targeted therapies have shown efficacy in tumors with specific driver mutations, such as *EGFR*, *ALK*, or *ROS1*, but tumors can sometimes develop secondary or even tertiary mutations that render these treatments ineffective (Facchinetti et al., 2018). However, osimertinib and other third- and second-generation therapies can counteract this (Xiang et al., 2024). Another example is seen with *EGFR* T790M mutations and C797S substitutions, which confer resistance to first-generation EGFR TKIs such as erlotinib, but second-generation EGFR TKIs can often counteract this (Kang et al., 2024; Borghaei et al., 2015; Zhuang et al., 2019). While osimertinib is an effective third-generation EGFR TKI, resistance mediated by the C797S mutation has emerged as a limitation, prompting research into fourth-generation EGFR TKIs to overcome it. These next-generation inhibitors aim to either bind to EGFR via alternate mechanisms, such as allosteric inhibition, or selectively target mutant receptors, thereby improving safety and efficacy.

Resistance mechanisms to targeted therapies can be broadly categorized into genetic and non-genetic adaptations (Ou et al., 2024). Genetic resistance involves secondary or tertiary alterations in target genes, which can directly impair drug binding or amplify target abundance (Johnson et al., 2022). This resistance mechanism explains cases involving EGFR TKIs (e.g., EGFR T790M and EGFR C797S) and MET amplification as a bypass pathway. Non-genetic resistance mechanisms include epigenetic reprogramming, TME remodeling, EMT, and lineage plasticity, most notably the histological transformation of NSCLC to SCLC (Sanchez-Cespedes et al., 2026; Ou et al., 2024). Distinguishing between these modes is essential for targeted secondary interventions rather than a broader, general combination approach.

Early preclinical and clinical studies suggest promising results in fourth-generation TKIs and may restore sensitivity in tumors that have become resistant to osimertinib (Corvaja et al., 2024). The main issue with therapeutic failure is adaptive resistance via feedback loops, in which inhibition of EGFR signaling activates bypass pathways, such as MET and KRAS, thereby restoring downstream MAPK and PI3K signaling despite pharmacological blockade. This creates a temporal response in which tumor signaling shifts under pressure, enabling survival through pathway reprogramming rather than a single mutation. Immunotherapy resistance also arises through immune evasion mechanisms, including loss of antigen presentation, upregulation of alternative immune checkpoints (e.g., LAG-3), and suppression of T cell infiltration and cytokine activity.

To address resistance challenges, combination therapies have emerged as a solution. One example of this is the exploration of EGFR-TKI and PD-1/PD-L1 blockade to overcome adaptive resistance and restore immune detection of tumor cells (Kang et al., 2024; Shen et al., 2018) (Figure 5). EGFR mutations can alter immune signaling by reducing antigen presentation which hinders the immune response to PD-1/PD-L1 blockade. Combining EGFR inhibition with targeting bypass pathways, such as MET, can help intercept tumor signaling before resistant clones become dominant in the TME. Targeting immunosuppressive populations such as TAMs and MDSCs enhances therapeutic synergy by relieving immune suppression and restoring cytotoxic T cell activity, thus amplifying the effectiveness of combination therapies beyond effects (Figure 5) (Wong et al., 2022; Altorki et al., 2019; Khusnurrokhman and Wati, 2022; Riabov et al., 2014).

**FIGURE 5:**
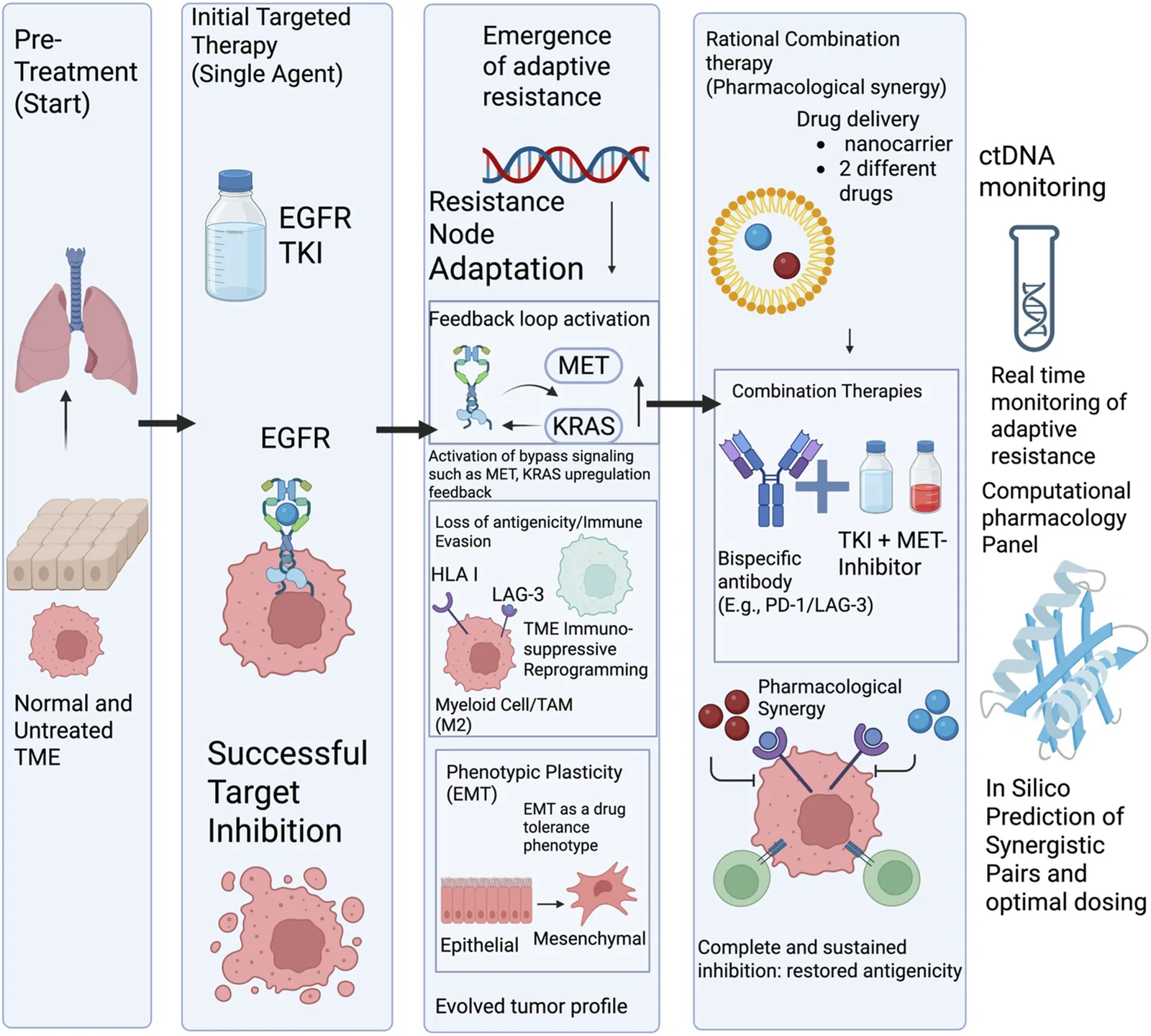
Pharmacological intervention of adaptive resistance in NSCLC. Upon NSCLC diagnosis, the malignant cells within the TME (panel 1) may be targeted with a monotherapy, such as an EGFR-TKI inhibitor (panel 2). While primary targeted inhibition is transiently successful, adaptive resistance can emerge (panel 3) via activation of feedback loops (MET/KRAS bypass signaling), immune evasion (LAG-3 upregulation and macrophage reprogramming), and phenotypic plasticity (EMT). A combination strategy with pharmacological synergy can interfere with such adaptive defenses and help achieve target inhibition, restore antigenicity, and reprogram the TME (panel 4). Examples include coordinated delivery systems (nanocarriers) and multiple blockade strategies (e.g., bispecific antibodies or TKI + MET inhibitors). Strategies, including ctDNA monitoring of tumor progression and *in silico* predictions, can help inform personalized treatment approaches to enhance patient outcomes (panel 5) (Kang et al., 2024; Xiang et al., 2024; Shen et al., 2018; Khaddour et al., 2021). Created in BioRender. BioRender.com/1jjxnvg.

Clinically, liquid biopsies have been essential tools for monitoring treatment and addressing emerging resistance. By detecting circulating tumor DNA (ctDNA), clinicians can noninvasively monitor tumor burden and adaptations in real-time, as ctDNA is released when tumor cells undergo apoptosis (Khaddour et al., 2021; Li et al., 2023a). Furthermore, adaptive trials have enabled rapid testing of treatments tailored to evolving tumor cells, helping to control tumor progression (Bray et al., 2024; Borghaei et al., 2015). Overall, resistance in lung cancer is best understood as a dynamic, feedback-driven evolutionary system requiring extensive pharmacological strategies designed for true synergy rather than additive inhibition of isolated pathways.

### 5.3 Managing toxicity and adverse events

The increasing use of targeted and immunotherapies has significantly improved outcomes in lung cancer treatment; however, these developments cause new challenges relating to toxicity. Immune checkpoint inhibitors can cause immune-related adverse events, including increased skin toxicity and pneumonitis (Marrone et al., 2016). Endocrinopathy is an additional concern which may necessitate discontinuation of treatment or require additional immunosuppressive therapy (Deshmukh, 2023; Siddiqui et al., 2025). Targeted therapies, although more selective, also carry risks, as evidenced by gastrointestinal toxicity associated with trophoblast cell surface antigen-2-directed antibody-drug conjugates, sacituzumab govitecan, for treating lung cancer (Stumpo et al., 2025).

To mitigate these effects, identifying biomarkers that can prevent or reverse toxicity immediately after treatment, such as HLA genotypes or baseline cytokine levels, is key for effective and beneficial treatments for high-risk patients (Wang et al., 2022a; Chennamadhavuni et al., 2022). Furthermore, nanomedicine and nanodrug delivery systems are currently under development to minimize cytotoxicity during therapy (Sharma et al., 2023; Li et al., 2024; Garg et al., 2019) (Figure 6). One of the major mechanisms by which nanomedicine reduces toxicity is the enhanced permeability and retention (EPR) effect in which nanoparticles accumulate in tumor tissue due to their “leaky” vasculature. This selective accumulation enables localized high drug concentration while minimizing systemic exposure, thereby preserving therapeutic efficacy while reducing off-target toxicity. In addition to reducing treatment toxicity, biomarkers also play a crucial role in predicting therapeutic response and guiding personalized treatment strategies in precision oncology.

**FIGURE 6:**
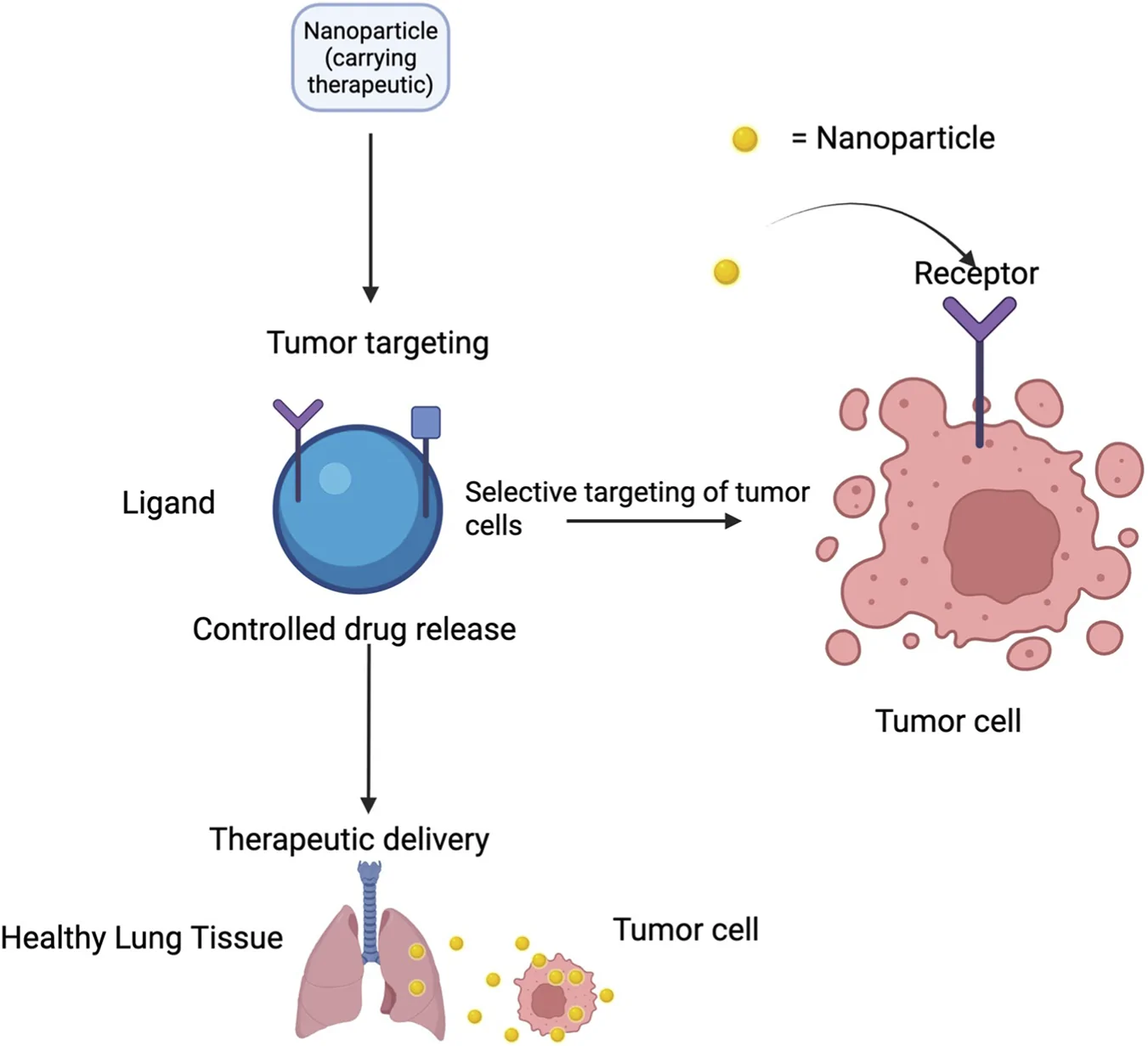
Application of nanomedicine in lung cancer: A flow chart describing how nanomedicine works in lung cancer therapy. The nanoparticle enhances drug release and cancer cell eradication with reduced toxicity (Sharma et al., 2023; Alshammari et al., 2023; Rawal and Patel, 2021). Created in BioRender. BioRender.com/89ijcr7.

### 5.4 Addressing tumor heterogeneity and evolution

Tumor heterogeneity, the differences in genomic, resistance, and immunologic profiles across tumor regions or between metastatic sites, is a key driver of resistance and tumor progression. Intratumor diversity limits the efficacy of monotherapies and could foster tumor evolution under selective pressure (Kang et al., 2024; Borghaei et al., 2015; Khaddour et al., 2021; Sun and Yu, 2015).

Clonal evolution, as observed through longitudinal genomic tracking, can reveal that resistant clones exist at low frequencies but become dominant following treatment (Burrell and Swanton, 2014). Studies using single-cell RNA sequencing show the complexity of tumor subpopulations in the TME, which can shift under therapeutic pressure, such as through mutations (Khaddour et al., 2021; He et al., 2021). Additionally, preclinical platforms such as organoids and PDX are essential for modeling evolutionary tumor dynamics and evaluating combination strategies designed to achieve pharmacological synergy across heterogeneous tumor populations (Sharma et al., 2023; Alshammari et al., 2023; Huo et al., 2020).

Clinically personalized treatment strategies that allow adjustment based on biopsy or ctDNA profiling are currently being tested to determine whether they can overcome clonal evolution (Vidal et al., 2020). Combination therapies allow targeting multiple resistance pathways when they occur simultaneously, such as KRAS and ALK, thereby extending therapeutic efficacy (Kang et al., 2024; Borghaei et al., 2015). Combination therapies are therefore essential not only for broader coverage but also to achieve synergistic suppression of multiple evolutionary escape pathways, including KRAS and ALK mechanisms.

## 6 Future directions in lung cancer treatment

### 6.1 Identifying predictive biomarkers

Precision oncology is the effective use of predictive biomarkers to optimize patient therapy based on their individual tumor. Currently, PD-L1 expression is used to guide PD-1/PD-L1 inhibitors; however, its predictive value is inconsistent across patients due to variability in testing. An alternative would be to pair it with plasma cells, given their predictive value for improving overall survival in NSCLC with immunotherapy (Lim et al., 2023). TMB and microsatellite instability are additional biomarkers for tracking tumor mutations and can identify potential candidates for immunotherapy (Xiang et al., 2024). Other emerging biomarkers include TIL activity, cytokine signatures, and co-stimulatory molecules, which reflect immune activity within the TME and correlate with treatment response (Wang et al., 2022a; Derraze et al., 2025).

TME profiling can assess the interactions between suppressive and stimulatory responses of immune cell populations, such as neutrophils, dendritic cells, and macrophages, providing deeper insights into tumor immunogenicity, immune responses, and resistance patterns (Lim et al., 2023). Non-invasive strategies, such as liquid biopsies, have enhanced biomarker discovery by enabling real-time monitoring of tumor progression (Khaddour et al., 2021; Li et al., 2023a). When integrated with AI data analysis, these approaches could drive patient stratification and personalize therapy further (Khaddour et al., 2021; Abdelhamid et al., 2026). Importantly, machine learning models can integrate multi-omic biomarker profiles to predict optimal pharmacological synergies and identify patients most likely to benefit from specific combination therapies.

### 6.2 Nanotechnology-directed drug delivery systems

Nanotechnology has the potential to overcome long-standing challenges in lung cancer drug delivery. Currently, engineered nanoparticles and liposomes enhance pharmacological properties, including circulation time, solubility, and tumor-specific accumulation via EPR and enhanced permeability (Sharma et al., 2023; Alshammari et al., 2023). Furthermore, nano-drug delivery strategies help minimize off-target cytotoxic effects on non-malignant cells (Sharma et al., 2023). Bioderived ligand-conjugated nanosystems further enable tumor cell selectivity while reducing toxicity in preclinical trials (Rawal and Patel, 2021).

In preclinical settings, nanocarriers that encapsulate chemotherapeutics (e.g., paclitaxel) or molecular inhibitors (e.g., gefitinib) have been shown to exhibit improved tumor uptake and reduced toxicity in mouse models (Sharma et al., 2023; Alshammari et al., 2023). Several nanoformulations have been utilized in clinical trials for NSCLC, including liposomes, micelles, and albumin-bound combinations (Holder et al., 2023). For instance, nanoparticle albumin-bound paclitaxel (nab-paclitaxel) plus carboplatin treatment significantly increase the overall response rate and decreased several off-target effects compared to the solvent formulation (Socinski et al., 2012). Nab-paclitaxel has been further assessed for safety and efficacy in patients with advanced NSCLC (Yoneshima et al., 2021). Further formulations also enable co-delivery of drugs, such as stimuli-responsive carriers for controlled release triggered by enzymes or pH, which is crucial given the acidity of the lung TME (Sharma et al., 2023; Hosonuma and Yoshimura, 2023). A major emerging direction is nanomedicine-enabled pharmacological synergy in which co-encapsulated drugs are released to simultaneously inhibit pathways and maximize synergistic therapeutic effects at the tumor site (Chen et al., 2023). These platforms help maintain fixed, optimal stoichiometric ratios of multiple therapeutic agents throughout circulation until drug release (Khade et al., 2025). By coordinating when and where treatments are delivered, this approach allows for dual-pathway suppression directly within malignant environments, maximizing synergistic efficacy while minimizing several off-target toxicities (Mousavi-Kiasary et al., 2025).

Clinically, nab-paclitaxel is already FDA-approved, and other agents are currently in early-phase trials (Villaruz and Socinski, 2016). Further efforts aim to combine nanomedicine with immunotherapy to restore the TME to its normal state, enhancing antigen presentation and further T cell infiltration (Kim et al., 2021). Nanocarriers are also being explored for siRNA delivery, which offers gene-silencing potential for driver mutations and resistance pathways (Sharma et al., 2023).

### 6.3 Artificial intelligence and machine learning in patient selection and drug discovery

Artificial intelligence (AI) is rapidly transforming lung cancer research and can support clinical practice by enhancing analysis, visualization, and automation of many variables in the treatment process. In drug discovery, AI-driven molecular docking and models are accelerating the identification of compounds with high affinity for oncogenic proteins (Gao et al., 2023; Jiménez-Luna et al., 2021; Ocana et al., 2025). Machine learning has also been used to simulate resistance development, drug side effects, and other potential combinations of therapies that could delay or prevent tumor progression in many clinical settings (Khaddour et al., 2021; Gao et al., 2023).

In clinical settings, AI algorithms that analyze radiologic imaging, such as CT scans, serve as noninvasive tools for predicting biomarker expression, including PD-L1 and TMB, thereby supporting immunotherapy planning before treatment (Xiang et al., 2024). Electronic health record-based models are being used to assess risk and allow customized treatment schedules (Pinsky, 2021; Rajkomar et al., 2018). Multimodal AI systems integrate imaging, genomic, transcriptomic, and clinical datasets to model tumor behavior as a dynamic system that can predict optimal pharmacological synergies in real-time. These systems could support oncologists in selecting the optimal therapeutic strategy for each patient (Khaddour et al., 2021; Pinsky, 2021; Vale-Silva and Rohr, 2021).

Additionally, these systems aim to predict therapeutic synergy and resistance. With new modeling changes to account for tumor evolution, AI can help identify adaptive resistance mechanisms earlier in treatment regimens. Algorithms enable simulations of how tumors respond to specific therapies, allowing optimization of coordinated drug release to suppress complementary signaling networks. This approach may enable better identification of patient-specific information, such as therapeutic windows, and reduces side effects, including nanotoxicity. As technology advances, AI platforms can play a larger role in guiding pharmacological synergy and precision medicine, enabling maximal long-term efficacy and overcoming resistance mechanisms in lung cancer.

## 7 Conclusion

Transforming the current paradigm of lung cancer treatment requires moving away from traditional therapies toward precision oncology, which uses specialized, multi-targeted approaches. Treatment of lung cancer has been revolutionized in the past few years due to new developments with targeted therapy and immunotherapy. Targeted therapies have drastically improved outcomes by focusing on specific oncogenic drivers (such as *EGFR*, *ALK*, and *KRAS* mutations), yet they remain limited by the likelihood of acquired secondary or tertiary mutations and the reactivation of alternate pathways. In addition, immunotherapies and immune checkpoint inhibitors effectively counteract immune evasion and restore cytotoxic T-cell function, but their monotherapeutic efficacy is often limited by intratumoral heterogeneity and an immunosuppressive tumor microenvironment. To overcome these adaptive resistance networks, combination therapies leverage true pharmacological synergy. By pairing direct oncogenic signaling blockades with immune reactivation and optimizing delivery via advanced nanocarriers, combination strategies enhance drug delivery, reduce systemic toxicity, and simultaneously target multiple resistance pathways. However, the future implementation of these complex regimens faces several limitations, including severe immune-related or gastrointestinal adverse events, tumor clonal evolution, data confidentiality issues, and a lack of racial diversity in the genomic datasets required to predict patient-specific drug responses. Moving forward, resolving these challenges requires integrating multi-omic biomarker profiling, liquid biopsy-based ctDNA tracking, and multi-modal artificial intelligence to safely and equitably optimize personalized therapeutic windows for lung cancer patients. Continued research and ethical consideration of these developments could lead to an optimistic outlook for a safer, more effective treatment of lung cancer.
